# Dengue infection during pregnancy in Burkina Faso: a cross-sectional study

**DOI:** 10.1186/s12879-019-4587-x

**Published:** 2019-11-27

**Authors:** Kongnimissom Apoline Sondo, Adama Ouattara, Eric Arnaud Diendéré, Ismaèl Diallo, Jacques Zoungrana, Guelilou Zémané, Léa Da, Arouna Gnamou, Bertrand Meda, Armel Poda, Hyacinthe Zamané, Ali Ouédraogo, Macaire Ouédraogo, Blandine Thieba/Bonané

**Affiliations:** 1 Joseph Ki-Zerbo University, Ouagadougou, Burkina Faso; 2 Department of Infectious Diseases (Yalgado Ouedraogo Teaching Hospital), Ouagadougou, Burkina Faso; 3 Obstetrics and Gynecology Departement (Yalgado Ouedraogo Teaching Hospital), Ouagadougou, Burkina Faso; 4 Higher Health Science Institute, Polytechnic University of Bobo-Dioulasso, Bobo-Dioulasso, Burkina Faso; 5 Health Science Research Institute of Ouagadougou (Biomedical department), Ouagadougou, Burkina Faso

**Keywords:** Dengue fever, Pregnant woman, Burkina Faso

## Abstract

**Background:**

Dengue fever is prevalent in the world; in recent years, several outbreaks occurred in West Africa. It affects pregnant women. We aimed to assess the consequences of dengue fever on pregnant women and their fetuses during dengue epidemic in Burkina Faso.

**Methods:**

We conducted a cross-sectional study from November 1, 2015 to January 31, 2017 in 15 public and private health facilities in Ouagadougou, using secondary data. Immunochromatographic rapid test Duo detecting specific antibodies, immunoglobin M/G and /or dengue non structural antigen1 virus was used to diagnose dengue cases.

**Results:**

Out of 399 (48%) women registered during the study period, 25 (6%) were pregnant. The average age of pregnant women was 30 years, with 18 and 45 years as extremes. The main symptoms were fever (92%) and headache (92%). Nine patients (36%) had severe dengue characterized by bleeding (16%), neurological symptoms (16%) and acute respiratory distress (8%). Eight (32%) of the 25 women had early miscarriage and 8 (32%) women gave birth to viable fetuses. Among those with viable babies, 5 (20%) presented post-partum hemorrhage and 3 (12%) presented early delivery. The main fetal complications included 3 cases of acute fetal distress (12%). One case of maternal death (4%) and 4 cases of neonatal mortality (44.5%) were notified.

**Conclusion:**

Dengue fever occurring during pregnancy increases maternal and neonatal mortality. Its severe complications require specific monitoring of pregnant women until delivery.

## Background

Dengue is an arboviral infection transmitted by mosquitos of the genus *Aedes* [1, 2]. The incidence of dengue has increased by a factor of 30 over the past 5 decades, with the emergence of many new affected countries [2]. About 2.5 billion people live in endemic areas and an estimated 50 million people are infected each year [1–3]. The disease can affect anyone but pregnant women are more at risk. The most common clinical symptom of dengue is fever that can cause abortion or early delivery. Dengue-related thrombocytopenia increases the risk of bleeding during pregnancy or at delivery, and therefore leads to higher maternal mortality rate.

Studies on the reciprocal influence between dengue and pregnancy are quite rare. The fetal consequences of dengue are neither well understood nor well documented [4]. Few data on dengue during pregnancy exist. Authors reported some cases in Asia, Europe or Latin America [1, 5, 6]. In sub-Saharan Africa, it was urgent to assess the extent of the disease. In Burkina Faso as in other sub-Saharan countries, dengue cases have been reported since 2013, with an outbreak in 2016. The objective of this survey was to describe the socio-epidemiological, clinical, biological aspects, and the evolution of dengue during pregnancy in Ouagadougou.

## Methods

This was a descriptive cross-sectional study involving 15 health facilities in the city of Ouagadougou: 4 District hospitals and 3 Teaching hospitals with packages of healthcare services corresponding to the secondary and tertiary levels of the health pyramid in Burkina Faso. In addition, eight private health facilities were selected according to their capacity of diagnosing dengue to be part of the study. We obtained the authorization of the General Director of Healthcare in Ouagadougou for data collection. The diagnosis of dengue was performed using rapid diagnostic tests (RDTs), which are immuno-chromatographic tests detecting Non Structural Antigen 1 (NS1Ag) and immune-globulin M and G (IgM and IgG). We used the World Health Organization classification of dengue (WHO 2009) to classify the women according to the severity of dengue.

The study population consisted of all the patients (male and female) diagnosed with dengue in these healthcare centers from November 1, 2015 to January 31, 2017, using dengue RDTs.

The sample consisted of all the pregnant women treated for dengue in these healthcare centers. The inclusion criteria were the positivity of the NS1 antigen and/or IgM and/or IgG. Isolated IgG positivity was considered a serological scar of dengue but we could not titrate this antibody.

Serotype 2 of dengue virus (DENV-2) was identified by Polymerase chain reaction (PCR) in the Pastor Institute of Dakar, as the causal agent of the epidemic during the study period [7].

The diagnosis of malaria was performed using malaria RDTs Histidine Rich Protein 2 and / or blood smears.

The data were collected from the consultation records, laboratories and the clinical records of the patients. The collected data were analyzed using EPI INFO version 3.5.

## Results

### Socio-epidemiological characteristics of pregnant women

**Fig. 1:**
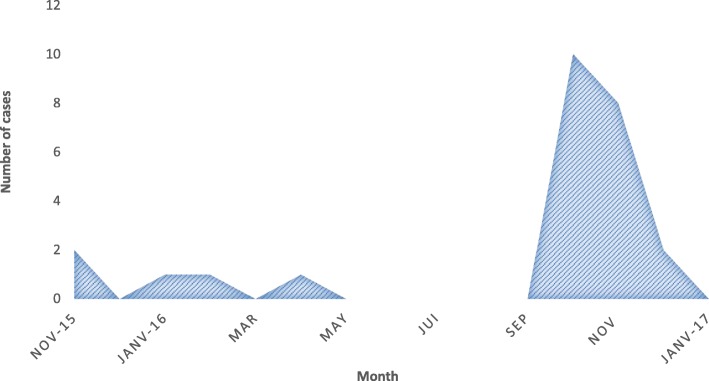
Distribution of dengue cases in pregnant women by month

In this study, 835 patients were screened for dengue, using rapid diagnostic tests (RDTs). Among them, there were 399 women (48%), including 25 (6.5%) pregnant women. Sixty-eight percent of the pregnant women were between the ages of 25 and 35 years. The average age of pregnant women was 30 years with 18 and 45 years as extremes, and 92% of pregnant women lived in Ouagadougou. Forty four percent (44%) had university degrees and 36% high school level. Thirty-wo percent (32%) were public and private sectors workers. Students accounted for 24%, as did housewives, and 20% of women were informal sector workers. Dengue frequency distribution during the study period showed a peak in October with 10 cases (40%), which corresponds to the peak in all the patients. Eight cases (32%) were notified in November 2016 (Fig. 1).

### Clinical and biological characteristics of pregnant women

Among the pregnant women diagnosed with dengue, 8 were in their first trimester of pregnancy, 8 in their second trimester and 9 in their third trimester.

The reported clinical symptoms were fever (92%), asthenia (64%) and painful symptoms (84%) including headaches (92%), arthralgia (72%) and lumbago/myalgia (64%). Hemorrhagic signs (32%) included epistaxis (24%) and metrorrhagia (16%). Four women (16%) presented neurological symptoms such as consciousness disorders (coma stage I = 1 case and coma stage II = 3 cases). Two cases of malaria-dengue co-infection were notified. The clinical characteristics of women are shown in Table 1.

**Table 1:** Distribution of cases according to clinical aspects

Clinical aspects	Frequency (%)
Stage of pregnancy	
First trimester	08 (32)
Second trimester	08 (32)
Third trimester	09 (36)
Clinical signes	
Fever	23 (92)
Painfull signs	21 (84)
Headaches	23 (92)
Arthralgia	18 (72)
Myalgia	16 (64)
Abdominal pain	12 (48)
Retro-orbicular pain	04 (16)
Asthenia	16 (64)
Vomiting	14 (56)
Hemorrhagic signs	08 (32)
Epistaxis	06 (24)
Metrorrhagia	04 (16)
Gingivorragia	03 (12)
Other^a^	04 (16)
Neurologics signs	04 (16)
Comatose stage II	02 (8)
Comatose stage I	02 (8)
Jaundice	03 (12)
Hépatomegaly	03 (12)
Dyspnea	02 (8)
Oligoanuria	02 (8)
Plasma leak (pleurisy)	01 (4)
Lithiasis	01 (4)

^a^ = Hematemesis, hematuria, melena, hemorrhagic at the injection site

Twenty-one of the 25 pregnant women (84%) had early dengue with positive NS1Ag. Sixteen pregnant women (64%) presented with primary dengue and 6 others (36%) with secondary dengue fever. Three patients were carriers of IgG alone.

Fifteen pregnant women (52%) had anemia with hemoglobin level less than 10 g/dl. Two of them were transfused with packed red blood cells. Thrombocytopenia defined as platelet count less than 150,000 / mm3, was observed in 12 patients (48%). Six of them had platelet count less than 50,000 / mm3 and 5 women received transfusion of platelets. Six women (24%) had elevated levels of aspartate aminotransferase (ASAT) ranging from 2 to 15 times the normal value. Two patients had a serum creatinine higher than 120 μmol/l.

Sixteen women (64%) had dengue fever with warning signs while 9 women (36%) had severe dengue fever, according to the 2009 WHO classification.

### Maternal and fetal consequences

#### In mothers

Maternal complications were present in 6 cases. There was early delivery in 3 cases (12%) and intra partum hemorrhage in 5 cases (20%). Three women gave birth vaginally while 3 others underwent caesarean section. Two women expelled macerated fetuses. Seventeen women were always pregnant while they were discharged, and 8 women gave birth, with one of them having twins. A death was recorded in a context of liver failure and postpartum hemorrhage, which stands for 4% of lethality. Lethality was 12.5% among those who gave birth.

#### In fetuses and newborns

Fetal complications were observed in 6 cases including 3 cases (12%) of fetal distress and 3 cases (12%) of prematurity. Four cases of neonatal deaths (44.5%) were notified, 3 of which were due to prematurity and one case to neonatal infection. Women and fetal consequences are summarized in Table 2.

**Table 2:** Maternal and perinatal outcomes

Item	Frequency (%)
Mother outcome	
Death	1 (4)
Exit without medical advice	1 (4)
Maternal death (*n* = 8: known outcome)	1 (12,5)
Pregnancy outcome	
Normal pregnancy	17 (56)
Delivery ^a^	8 (36)
Normal vaginal delivery (living newborn)	3 (12)
Preterm delivery (macerated dead fetus)	2 (8)
Caesarean section	3 (12)
Living newborns (*n* = 9 birth)	5 (55.5)
Dead (n = 9 birth)	4 (44.5)
Death in-utero	2 (8)
Perinatal death	2 (4)
Maternal complications	6 (24)
Hemorrhagic delivery^b^	5 (20)
Preterm delivery	3 (12)
Dynamic dystocia	2 (8)
Fetal complications	5 (20)
Fetal distress	3 (12)
Prematurity	3 (12)
Néonatal infections	2 (8)

^a^ = Eight deliveries with 9 children: one twin birth

^b^delivery bleeding (4) and parietal hematoma after cesarean section = 1

## Discussion

During the study period, 25 pregnant women were infected by dengue virus in Ouagadougou. The monthly epidemiological curve showed a maximum number of cases in October and November. The proportion of pregnant women with dengue was probably underestimated during our study as we only described the cases present in our database and as the survey was conducted in facilities not frequented by all pregnant women. Dengue surveillance should be instituted in pregnant women in order to distinguish it from malaria, since the two diseases share many common symptoms. The patients’ average age was 30 years, close the result of a descriptive survey conducted in Rio de Janeiro [8], and lower than the results found in other series in French Guiana [9] and Malaysia [5]. The disparity in the age of pregnant women could be justified by the fact that the average age of first maternity differs from one country to another, by sociocultural differences, and development level differences.

Unlike the common situation in developing countries where most of women suffering from infectious diseases are unemployed, housewives or farmers, most of pregnant women in our series were public and private sectors workers. Therefore, most of them could afford for the rapid diagnostic test cost (15 to 25 euros). This fact shows that pregnant women from low socioeconomic categories (housewives, farmers, unemployed) suffering from dengue but unable to afford for RDTs cost could not be diagnosed. The dengue frequency in pregnant women was underestimated, regarding the 2016 dengue outbreak in the city of Ouagadougou [7].

Out of the 25 pregnant women infected by dengue in our study, 84% were at phase of viremia. In Colombia, a reverse situation was observed in two studies with an acute viremic phase of 29.9 and 27.3% [6, 10]. In our context, dengue diagnosis was performed early compared to other authors’ results because of the epidemic context. About 9 cases of probably secondary dengue were observed, showing that these patients had caught a previous infection by dengue virus; that could explain the high proportion of severe mother and child complications (24 and 20% respectively). On the other hand, this high proportion of complications in Sub Saharan Africa contrasts with the results reported by some authors who suggested that being black was a protective factor and could explain the low proportions of severe dengue notified in African series [7, 11, 12].

In our study, the reported cases showed an upward distribution according to the trimester of pregnancy, with a doubling of the number of cases from the 1st to the 3rd trimester. Similar findings were reported in Colombia [13]. However, some authors reported higher infection frequency during the first trimester of pregnancy (45.4%) [10]. Carles in French Guiana found a higher incidence of the infection during the second trimester (40.9%) [14]. The period of occurrence of dengue seems to determine the types of complications, to the point that pregnant women infected during their first trimester had higher risk of miscarriage [3, 15]. When the infection occurred during the last trimester, the risk of low birth weight, premature labor and vertical transmission seemed to be higher [3, 8, 13, 16]. However other factors could explain the low birth weight as well as the prematurity. In our series, the evolution and the outcome of the pregnancies of women infected in their first and second trimester were not taken into account. However, out of 9 women in their third trimester of pregnancy, we observed 8 deliveries during the acute stage of dengue, with delivery bleeding 5/25 (20%) and prematurity 3/8 (12%). Almost half (45.5%) of the 9 newborns died. In the literature, there is a high percentage of threating preterm delivery, as shown in series conducted in Malaysia (50%) [5] and French Guiana (55%) [13]. A study in Cuba reported a risk of preterm delivery 3.7 times higher than in our series [17]. A specific management of pregnant women with dengue fever, especially in the last trimester, could reduce the risk of prematurity and prevent the risk of bleeding during delivery.

In our study, we recorded only 3 cases of Caesarean section out of the eight deliveries. Most of the studies reported as many cases of Caesarean section as natural deliveries; for instance, Alvarenga and Leon reported 50 and 53.8% respectively [8, 18]. Considering the high risk of bleeding in pregnant women with dengue, caesarean section practice would reduce the incidence of postpartum bleeding.

We recorded one maternal death out of 25 cases. Maternal mortality is significant in most studies: 17/78 in Sudan, 2/13 in Rio de Janeiro, and 3/16 in South Asia [5, 8, 19]. The recorded case of death in our series occurred in a context of complications, similarly to the situation reported by Machado in Brazil who found that pregnant women were 3.4 times more susceptible to catch severe dengue, and that dengue-related mortality in pregnant women was higher than in non-pregnant women [20, 21]. Therefore, any pregnant woman diagnosed with dengue must be considered as having a high risk of developing severe dengue. Such women require rigorous medical surveillance.

## Conclusion

Our study showed that pregnant women are at high risk of complications when they catch dengue fever. The usual bleeding during delivery may be aggravated by this disease. On the other hand, some of the complications noted in this study cannot be exclusively attributed to dengue fever. Nevertheless, these complications should be taken into account in order to prevent the risk of bleeding and preterm delivery.

## Data Availability

The data used during the study are available from the corresponding author.

## Abbreviations

ASAT: Aspartate aminotransferase
IgG: Immunoglobin G
IgM: Immunoglobin M
NS1Ag: Non Structural Antigen 1
PCR: Polymerase Chain Reaction
RDTs: Rapid Diagnostic Tests
WHO: World Health Organization
